# The relationship between team ability and home advantage in the English football league system

**DOI:** 10.1007/s12662-021-00721-x

**Published:** 2021-05-26

**Authors:** Girish Ramchandani, Robbie Millar, Darryl Wilson

**Affiliations:** Academy of Sport and Physical Activity, Sheffield Hallam University, Sheffield, UK

**Keywords:** Team sports, Soccer, England, Game location, Performance

## Abstract

The existence of home advantage (HA) has been found in a variety of team sports including football. There is a paucity of research on the relationship between team ability and HA in domestic football leagues and the findings of previous studies are inconclusive. Using longitudinal data from the top four football divisions in England, this study investigates the influence of team ability on the HA of teams. The data collected for this study spans 24 seasons from 1995/96 to 2018/19 and includes 48,864 matches from the English Premier League (*n* = 9120), the Championship (*n* = 13,248), League One (*n* = 13,248) and League Two (*n* = 13,248). Team ability was interpreted in two ways: (1) the division in which teams play and (2) their league table position within each division. For both the divisional and positional analysis, HA was calculated as the ratio of home points to total points achieved by teams in each season under review. Evidence of a statistically significant HA was found in all four divisions and for teams of all abilities within each division. Small but statistically significant differences in HA were observed between divisions and between high, moderate and low ability teams within divisions.

## Introduction

The phenomenon of home advantage (HA), where home teams in sports competitions win over half of the games played under a balanced home and away schedule (Courneya & Carron, 1992), has received widespread attention from researchers. The existence of HA has been documented in professional team sports such as domestic football leagues (Pollard, Prieto, & Gomez, 2017a), single-sport events such as the IAAF World Indoor Championships (Ramchandani & Wilson, 2020) as well as multisport events including the Olympic and Paralympic Games (Wilson & Ramchandani, 2018) and the Commonwealth Games (Ramchandani & Wilson, 2012). However, there are also studies that illustrate the absence of HA in specific sporting contests and even the prevalence of a disadvantage in some cases (Wright & Voyer, 1995). It has been suggested that, except for subjectively evaluated sports, HA is not a major factor in individual sports, and its role in individual sports is much less comparable to its role in team sports (Jones, 2013).

A meta-analysis by Jamieson (2010) concluded that the HA effect for soccer (association football) was significantly stronger than that of nine other sports (American football, baseball, hockey, basketball, cricket, Australian rules football/rugby, golf, tennis and boxing). A more recent and extensive study by Pollard et al. (2017a) examined HA between 15 different team sports using data from 165 different countries both for men’s and women’s competition. The HA found for football in the Pollard et al. (2017a) study was somewhat below its historical position relative to other sports. Within football, the existence of HA across national domestic leagues worldwide was illustrated by Pollard and Gomez (2014a). Incorporating 157 national domestic football leagues over six seasons between 2006 and 2012, this study showed that HA was present in all continents, but varied considerably between countries. A separate study by the same authors concluded that the HA effect was also evident in women’s football leagues throughout Europe, but the level of HA was lower than the corresponding men’s leagues (Pollard & Gomez, 2014b). The potential causes of HA in football include factors such as crowd effects, familiarity, referee bias, travel effects, territoriality, psychological factors and tactics (Pollard, 2008). However, as noted by Pollard et al. (2017a) determining how these factors operate and the way in which they affect performance is still unclear.

### HA and team ability

There is growing interest among researchers on the relationship between HA in football and team ability. The terms “quality” and “strength” are sometimes used interchangeably by researchers when referring to team ability. The performance of a team in a particular season depends on the quality of that team, the quality of its opponents and the size of the HA (Clarke & Norman, 1995). In other words, performance has two components, namely: quality and HA. If every team in a league enjoys the same level of HA, then performance is dependent on quality alone; however, if some teams have superior HA then their performance will be naturally enhanced. What this means is that HA is only relevant for the overall performance of a club if it is higher or lower than the average HA in its division (Peeters & van Ours, 2020). The literature on this aspect of HA can be categorised into two broad strands: divisional (inter league) HA and positional (intra league) HA. These strands of the literature are reviewed below.

#### Divisional HA

The hierarchical structure of domestic football leagues regulated through the system of promotion and relegation means that teams that feature in higher divisions are relatively stronger than teams that feature in lower divisions. Divisional HA is related to a specific division as a whole (e.g. the Premier League in England) and allows for making comparisons between different divisions in the same country (e.g. between the Premier League and the Championship in England) or between different leagues in two or more countries (e.g. between the Premier League in England and the Bundesliga in Germany).

Research on the effect of team ability on divisional HA in football has been conducted mainly from the perspective of the top two divisions in domestic football leagues and provided some mixed findings. Some studies have shown that HA is of a similar magnitude in the top two divisions. Pollard (2006) compared HA in the first and second divisions of five European domestic football leagues (Germany, England, France, Spain and Italy) across six seasons between 1996–97 and 2001–02 and observed that “very small differences” existed between the top two divisions in each of these countries. Subsequent research by Dosseville (2007), Seckin and Pollard (2008) and Sanchez, Garcia-Calvo, Leo, Pollard, and Gomez (2009) found no significant differences in the HA values between the top two divisions in France (between 2002/03 and 2004/05), Turkey (between 2002/03 and 2005/06) and Spain (between 1980/81 and 2006/07) respectively.

More recent studies have reported that HA in the second (lower) division of domestic football leagues is typically of a higher magnitude compared with the top (higher) division of domestic football leagues. HA has been shown to be significantly higher in the second division football leagues of both Brazil (Almeida, Oliveira, & Silva, 2011) and Iran (Pollard, Armatas, & Sani, 2017b). A more recent study by Leite and Pollard (2018) quantified HA for seven seasons from 2010/11 to 2016/17 of the top two divisions of domestic football leagues for 47 countries worldwide and concluded that HA was significantly more likely to be higher in the second division than the top division. They contended this may be due to players and referees in the top division being better trained to avoid being influenced by crowd support.

Few studies have sought to investigate the prevalence of HA in domestic football leagues beyond the top two divisions, which again have yielded diverse results. Nevill, Newall, & Gale (1996) examined HA in multiple divisions of English and Scottish football. Although their analysis was limited because it was based on a single season (1992/93), they found that higher HA was present in divisions with higher mean attendances. However, Pollard (2006) analysed data from all four professional football divisions in England across 12 seasons from 1992/93 to 2003/04 and found very little difference in the level of HA between them. A more recent study by Peeters and van Ours (2020), covering 45 seasons of English professional football from 1973/74 to 2017/18, also reported that absolute HA is about the same in all divisions, ranging from 0.59 to 0.64 in terms of points per match or from 0.44 to 0.46 in terms of goal difference.

Elsewhere in Europe, Armatas, Yiannakos, Seaton, and Rig (2013) found significantly higher HA appeared in the top division in the Greek Super League compared with the amateur divisions in Greece, suggesting the results could be associated with higher attendances and support of the home crowd. Conversely, a study of Portuguese football examining the level of HA in the professional league compared to semiprofessional and amateur leagues over a period of 11 seasons reported that HA was significantly lowest in the professional league (Almeida & Volossovitch, 2017).

#### Positional HA

Positional HA is concerned with individual teams. Teams’ final league table position is a function of their match outcomes during the course of a season (wins, draws and losses), which provides a composite measure of both their offensive (goals scored) and defensive (goals conceded) abilities. Heuer and Rubner (2014) note that team strength in football remains constant during the course of a season apart from short-time fluctuations. Hence, the final league table position in each season can be considered a reasonable proxy for team ability.

Teams of a higher ability are likely to win the majority of their matches at home and away, which means their HA would not be as significant as lower ability teams, who are inclined to focus on acquiring the majority of their points at home (Barnett & Hilditch, 1993; Bray, Law, & Foyle, 2003; Clarke & Norman, 1995). While this point makes sense intuitively, the investigation of HA from the perspective of positional team ability is underdeveloped. Allen and Jones (2014) analysed archival data from the first 20 seasons of the English Premier League and concluded that HA is greater in low ability teams than in high ability teams. Research by Liu, Garcia-De-Alcaraz, Zhang, & Zhang (2019) and Lago-Penas and Lago-Ballesteros (2011) found that superior and inferior teams did not experience the same HA in the Chinese Super League and La Liga respectively. Their findings confirm that a superior home team would be expected to win a higher percentage of games against inferior visiting teams, than against equally matched visitors.

Using a balanced panel of 65 clubs, which featured in one of the top four divisions of English football in every season over the period 1973/74–2017/18, Peeters and van Ours (2020) concluded that individual clubs differ substantially in the relative HA they enjoy. They found that HA fluctuated between 0.36 and 0.99 points per match (and between 0.27 and 0.71 in terms of goal difference). On the assumption that clubs that can spend more money should be able to have a better team, Peeters and van Ours (2020) utilised relative wage (expressed as the ratio of the club’s wage sum and the average wage sum in the division for the season) as a measure of quality, which did not have a significant effect on the relative HA of teams. However, they did not examine whether HA varies significantly according to the league table position of teams.

Building on this body of research and in view of the disparate and sometimes contradictory findings of previous studies, our study examines the relationship between team ability and HA from both a divisional (inter league) and positional (intra league) perspective in the top four divisions of football in England.

## Methods

### Scope of the study and data sources

This study covered 24 seasons from 1995/96 to 2018/19 of the top four football league divisions in England. The rationale for selecting this time frame for analysis was that the structure of the English football league system in terms of league branding, the number of teams in each division and the number of points awarded for a win remained consistent over these 24 seasons. The top division of football in the English football league system is the English Premier League (EPL), which has incorporated 20 teams per season in the time frame under review. Directly, below the EPL is the English Football League (EFL) which consists of three hierarchical divisions: the Championship, League One and League Two. Each EFL division consists of 24 teams per season. Matches in both the EPL and EFL are played on a balanced home and away basis. The overall sample included 48,864 matches from the EPL (*n* = 9120), the EFL Championship (*n* = 13,248), EFL League One (*n* = 13,248) and EFL League Two (*n* = 13,248).

The most comprehensive and well-researched conceptual framework that attempts to explain the HA phenomenon was developed by Carron and colleagues (Courneya & Carron, 1992; Carron, Loughhead, & Bray, 2005). According to this framework, performance outcomes influenced by game location can be measured at three levels. These three levels are the following: primary, relating to fundamental skill execution (e.g. possession, successful passes etc.); secondary, reflecting the scoring aspect of performance (e.g. number of goals scored or conceded); and, tertiary, representing the final outcome of the contest (win, draw or loss). In this study, we have analysed divisional and positional HA in relation to the tertiary measure of performance. Archival data on the final league tables that had a home and away split for the EPL and EFL for the 24 seasons were collated using publically available websites such as SoccerStats (https://www.soccerstats.com).

The sample details are presented in Table 1 including the number of matches (M) played per season, the number of home wins (HW), the number of draws (D) and the number of away wins (AW). In each season, team received three points for a win, one point for a draw and no points for a loss.

### Key variables

#### Home advantage

The overall approach to the calculation of HA in our study follows the method first proposed by Pollard (1986), which has been used widely in subsequent studies by different researchers. For any given league, HA is expressed as the number of points won by teams at their home fixtures during a season as a ratio of their total points achieved in that season, both at home and away, where a value in excess of 0.5 (or 50%) is indicative of HA (Leite & Pollard, 2018). We computed HA scores for every team in the EPL and EFL for each season under review (i.e. 20 EPL teams × 24 seasons plus 72 EFL teams × 24 seasons), giving us an aggregate sample of 2208 observations.

#### Team ability

Team ability was interpreted in two ways. Consistent with previous research (e.g. Armatas et al., 2013; Leite & Pollard, 2018; Pollard, 2006), the division in which teams played was assumed to be one indicator of team ability. In other words, it was assumed that team ability in the EPL was higher relative to the three divisions of the EFL. Similarly, within the EFL it was assumed that, in any given season, the ability of Championship teams was higher compared to teams in both League One and League Two, and also that League One teams were relatively stronger than those playing in League Two. For the divisional analysis of HA, we compared the mean divisional HA scores between the EPL and the three EFL divisions.

The second indicator of team ability was the final league table position of teams within each division at the end of any given season. This measure has been employed in previous research as a measure of team ability in English football (Allen & Jones, 2014). For the positional analysis of HA, we first calculated the HA scores associated with each position in the EPL (1 to 20) and the EFL (1 to 24 in each division) across the 24 seasons under review. Teams in the EPL and EFL were then classified into one of three groups according to their final ranking at the end of every season. The first group—high ability—consisted of the top 25% of teams, corresponding to the top five teams in the EPL and the top six teams in the EFL. The second group—moderate ability—comprised teams ranked 6–15 in the EPL and those ranked 7–18 in the EFL. Finally, the bottom 25% of teams, i.e. those ranked 16–20 in the EPL and those ranked 19–24 in the EFL, were in the low ability group. Our approach to grouping teams based on their league table position was informed by the work of Liu et al. (2019) and Lago-Penas and Lago-Ballesteros (2011).

Within our sample, there were 36 occurrences across the 24 seasons where teams had points deducted due to breaking league regulations (twice in the EPL, seven times in the Championship, 12 times in League One and 15 times in League Two), typically for financial mismanagement and clubs were deducted points for entering administration. Where such points’ deductions have occurred, we have reconfigured the relevant league tables so that a team’s league position is based entirely on their on-pitch performance.

### Statistical analysis

The data analysis was conducted using IBM SPSS Statistics (version 24). The prevalence of HA was tested using one-sample t‑tests by comparing the observed divisional and positional HA scores with a null value of 0.5 (indicating no HA). A one-way analysis of variance (ANOVA) was used to test whether there were any differences in HA between: (1) the top four football divisions in England and (2) teams of high, moderate and low ability within each division. Homogeneity of variances was checked using Levene’s test and suitable post hoc comparisons were made in each case. Spearman’s rank correlation was run to investigate the strength and direction of the relationship between league position and HA because league position was an ordinal variable.

## Results

### Divisional HA

The mean divisional HA scores and ANOVA results are summarised in Table 2. The HA scores for the EPL and all three EFL divisions were significantly greater than the neutral score of 0.5 (*p* < 0.001) as determined by one-sample t‑tests.

A one-way ANOVA confirmed a statistically significant difference in the HA scores between the top four football divisions in England (Welch (3, 1203.851) = 11.348, *p* < 0.001). A Games-Howell post hoc test for unequal variances showed that the size of the HA effect was significantly higher in the EPL compared with the EFL Championship (*p* < 0.05), EFL League One (*p* < 0.001) and EFL League Two (*p* < 0.001). The EFL Championship also had a significantly higher HA than EFL League Two (*p* < 0.05). No significant differences in divisional HA scores were observed between the EFL Championship and EFL League One (*p* = 0.331) or between EFL League One and EFL League Two (*p* = 0.645).

### Positional HA

The mean positional HA scores in the EPL and the three EFL divisions across the 24 seasons under review are shown in Table 3. There were significant positive correlations between league position and the corresponding positional HA scores within each division as per the correlation statistics presented in Table 4.

Table 5 shows the positional HA scores grouped into three hierarchical categories—high ability (top 25%), moderate ability (middle 50%) and low ability (bottom 25%)—according to the relative league table positions of teams in the EPL and EFL. A one-way ANOVA confirmed that HA scores differed significantly according to team ability within each of the top four football divisions in England (*p* < 0.01). Post hoc comparisons using a Games–Howell test (equal variances not assumed) revealed the following: (i) low ability teams had significantly higher HA compared with high ability teams in the EPL (*p* < 0.001), the EFL Championship (*p* < 0.001), EFL League One (*p* < 0.001) and EFL League Two (*p* < 0.01); (ii) low ability teams had higher HA compared with moderate ability teams in the EPL (*p* < 0.05) and EFL League One (*p* < 0.01); and, (iii) moderate ability teams had significantly higher HA compared with high ability teams in the EPL (*p* < 0.001), the EFL Championship (*p* < 0.001) and EFL League One (*p* < 0.05).

## Discussion

The aim of this study was to examine divisional and positional HA in the top four divisions of English football. Using data from 24 seasons from 1995/96 to 2018/19, our study has confirmed the prevalence of a statistically significant HA effect in the EPL and in all divisions of the EFL (Championship, League One and League Two). This finding in itself is not surprising and chimes with previous research on HA in domestic football leagues in England and across the world (e.g. Leite & Pollard, 2018; Peeters & van Ours, 2020; Pollard & Gomez, 2014). However, our study does provide some novel insights about the relationship between team ability and HA in English football.

When team ability was defined in terms of the division in which teams play, HA in the English football league system across the 24 seasons in our study fluctuated between 0.58 (58%) in EFL League Two and 0.61 (61%) in the EPL. Despite the relatively low volatility in our divisional HA scores (around three percentage points), we found a statistically significant difference in the magnitude of the divisional HA effect between the EPL and all three EFL divisions as well as between the highest and lowest divisions of the EFL.

It is possible that the significant differences observed in divisional HA might be related to some extent to the fact that EPL teams attract considerably larger crowds than teams in the EFL and that attendance in the EFL Championship is higher than in the two other EFL divisions. To illustrate this point, in the 2018/19 season the average attendance at EPL matches (38,168) was nearly twice the corresponding figure recorded for the EFL Championship (20,181), over four times higher than EFL League One (8741) and eight times higher than EFL League Two (4468). This assertion is given further credence by some studies that have demonstrated a positive association between crowd size and HA in football (Goumas, 2013, 2014a). Researchers have also shown that referees’ decisions in football matches can be influenced by the crowd to make decisions in favour of home teams (e.g. Goumas, 2014b; Pollard et al., 2017b; Seckin & Pollard, 2008) and that referees are responsible for some of the observed HA in the EPL (Boyko, Boyko, & Boyko, 2007; Lovell, Newell, & Parker, 2014). However, the effect of the crowd is difficult to establish conclusively because there were no significant differences in the divisional HA scores in the EFL between the Championship and League One or between League One and League Two. Therefore, there are likely to be other factors at play such as familiarity with local conditions, travel effects and territoriality (Pollard, 2008) that contribute to the occurrence of HA in the English domestic football league system.

Collectively, these findings indicate that while there appears to be some evidence of an association between team ability and divisional HA, this association is not necessarily linear in nature, which verifies previous research by Pollard (2006). What this also means is that there can be no implicit assumption that teams in a relatively higher (stronger) division would experience HA differently compared with those in a relatively lower (weaker) division. This view differs from other researchers who concluded that HA is likely to be of a greater magnitude at lower levels of competition in domestic football leagues (e.g. Almeida & Volossovitch, 2017; Leite & Pollard, 2018).

When team ability was defined more narrowly within each division according to where teams were positioned in the league table, the mean positional HA scores associated with all league table positions were found to be consistently greater than 50% (0.5) in all four divisions. This finding indicates that HA is prevalent in teams of all abilities in the EPL and the EFL. According to one previous study, teams finishing towards the lower end of the league table in the EPL exhibited a greater HA than those finishing towards the higher end of the league table (Allen & Jones, 2014). Aligned to this study, we found evidence of an inverse and statistically significant relationship between league position and HA in the EPL and the EFL. This finding was also supported by the ANOVA results comparing positional HA scores between teams of different abilities in the EPL and the EFL. It has been suggested that team quality is related to wages paid because richer clubs are able to attract better players (Peeters & van Ours, 2020). Because the evidence from our study illustrates that low ability teams tend to exhibit greater HA relative to high ability teams, we contend that the wage bill may be negatively associated with HA.

## Conclusion and future research

We found small but statistically significant differences in divisional HA between the EPL and all three EFL divisions and between the EFL Championship and the EFL League Two. We also found significant differences in positional HA between teams of different abilities in the EPL and EFL.

We have tested the influence of team ability on HA using the tertiary measure of performance, which relates to the final outcome of the contest (Courneya & Carron, 1992; Carron et al., 2005). Future research should investigate whether there are differences in divisional and positional HA in terms of primary and secondary performance measures relating to fundamental skill execution and the scoring aspect of performance. It would also be worthwhile to extend this investigation to domestic football leagues in other countries.

The last season of the EPL and EFL included in this study was 2018/19. The following season of these English football divisions, 2019/20, was interrupted in March 2020 due to the coronavirus disease 2019 (COVID‑19) outbreak. In May 2020, teams in EFL League One and EFL League Two voted to end their respective seasons with immediate effect. When the EPL and the EFL Championship seasons resumed in June 2020, teams were forced to complete their remaining fixtures behind closed doors with no spectators allowed in the stadium. Given that crowd support is one of the main factors thought to be responsible for HA in football (Pollard et al., 2017b), it would be worthwhile to examine the extent to which the COVID-19 induced absence of a supportive home crowd has impacted on HA in domestic football leagues in England and other countries.

**Table 1:** Sample overview

Season	Premier League				Championship				League One				League Two			
	M	HW	D	AW	M	HW	D	AW	M	HW	D	AW	M	HW	D	AW
1995/96	380	186	98	96	552	233	177	142	552	259	153	140	552	239	175	138
1996/97	380	162	119	99	552	262	150	140	552	264	155	133	552	256	148	148
1997/98	380	184	95	101	552	262	146	144	552	262	162	128	552	267	164	121
1998/99	380	169	115	96	552	250	163	139	552	243	146	163	552	253	147	152
1999/00	380	187	92	101	552	260	159	133	552	234	155	163	552	246	146	160
2000/01	380	184	101	95	552	247	148	157	552	242	151	159	552	273	157	122
2001/02	380	165	101	114	552	259	140	153	552	259	140	153	552	266	146	140
2002/03	380	187	90	103	552	247	143	162	552	236	150	166	552	231	167	154
2003/04	380	167	108	105	552	247	145	160	552	251	166	135	552	255	149	148
2004/05	380	173	110	97	552	235	162	155	552	244	149	159	552	249	162	141
2005/06	380	192	77	111	552	234	173	145	552	234	173	145	552	222	178	152
2006/07	380	182	98	100	552	266	123	163	552	249	139	164	552	247	142	163
2007/08	380	176	100	104	552	234	171	147	552	257	140	155	552	216	128	208
2008/09	380	173	97	110	552	239	162	151	552	236	137	179	552	228	164	160
2009/10	380	193	96	91	552	250	162	140	552	261	152	139	552	241	141	170
2010/11	380	179	111	90	552	246	148	158	552	249	137	166	552	225	168	159
2011/12	380	171	93	116	552	236	149	167	552	231	165	156	552	246	139	167
2012/13	380	166	108	106	552	246	145	161	552	222	149	181	552	227	150	175
2013/14	380	179	78	123	552	228	156	168	552	244	142	166	552	207	173	172
2014/15	380	172	93	115	552	228	158	166	552	223	145	184	552	244	139	169
2015/16	380	157	107	116	552	227	172	153	552	237	138	177	552	215	141	196
2016/17	380	187	84	109	552	262	130	160	552	248	154	150	552	221	143	188
2017/18	380	173	99	108	552	238	148	166	552	234	149	169	552	245	143	164
2018/19	380	181	71	128	552	240	163	149	552	230	147	175	552	244	148	160

*M* matches played, *HW* home wins, *D* draws, *AW* away wins

**Table 2:** Divisional home advantage (HA) in the English Premier League (EPL) and English Football League (EFL)

Division	*N*	HA		One Sample T Test (Test Value = 0.5)		Test of Homogeneity of Variances		Robust Test of Equality of Means	
		Mean	Standard Deviation	t	Sig	Levene Statistic	Sig	Welch^c^	Sig
Premier League	480^a^	0.61	0.08	29.476	0.000	4.594	0.003	11.348	0.000
Championship	576^b^	0.60	0.07	31.205	0.000				
League One	576^b^	0.59	0.07	28.957	0.000				
League Two	576^b^	0.58	0.08	25.375	0.000				

^a^20 teams × 24 seasons

^b^24 teams × 24 seasons

^c^Asymptotically F distributed

**Table 3:** Mean home advantage (HA) in the English Premier League (EPL) and English Football League (EFL) by league table position

League Position	Premier League			Championship			League One			League Two		
	HA^a^	t^b^	Sig	HA^a^	t^b^	Sig	HA^a^	t^b^	Sig	HA^a^	t^b^	Sig
1	0.57	6.603	0.000	0.56	9.563	0.000	0.54	3.767	0.001	0.56	5.049	0.000
2	0.57	6.875	0.000	0.56	11.134	0.000	0.57	7.474	0.000	0.57	4.42	0.000
3	0.57	5.933	0.000	0.55	3.806	0.001	0.56	5.298	0.000	0.56	4.689	0.000
4	0.58	8.874	0.000	0.58	8.977	0.000	0.58	6.62	0.000	0.58	5.575	0.000
5	0.59	7.793	0.000	0.57	7.683	0.000	0.56	5.557	0.000	0.56	3.909	0.001
6	0.59	6.649	0.000	0.59	6.177	0.000	0.59	8.557	0.000	0.58	7.153	0.000
7	0.60	6.921	0.000	0.58	5.458	0.000	0.59	7.219	0.000	0.58	6.874	0.000
8	0.60	7.205	0.000	0.61	6.999	0.000	0.59	7.295	0.000	0.60	7.973	0.000
9	0.63	9.815	0.000	0.58	6.439	0.000	0.59	6.956	0.000	0.57	6.088	0.000
10	0.60	5.43	0.000	0.62	11.808	0.000	0.61	9.317	0.000	0.56	3.909	0.001
11	0.61	9.4	0.000	0.59	7.249	0.000	0.57	6.579	0.000	0.56	3.994	0.001
12	0.64	6.779	0.000	0.61	9.455	0.000	0.59	5.271	0.000	0.62	7.437	0.000
13	0.64	11.281	0.000	0.58	4.813	0.000	0.56	3.304	0.003	0.57	4.197	0.000
14	0.62	5.728	0.000	0.59	5.955	0.000	0.58	5.292	0.000	0.58	5.057	0.000
15	0.60	5.649	0.000	0.60	8.088	0.000	0.59	5.843	0.000	0.58	4.675	0.000
16	0.63	7.257	0.000	0.60	6.442	0.000	0.60	7.006	0.000	0.60	6.735	0.000
17	0.64	7.539	0.000	0.63	12.1	0.000	0.57	4.514	0.000	0.60	5.793	0.000
18	0.64	5.99	0.000	0.64	8.333	0.000	0.58	4.276	0.000	0.58	3.804	0.001
19	0.67	9.905	0.000	0.60	7.336	0.000	0.60	7.296	0.000	0.60	6.363	0.000
20	0.62	5.291	0.000	0.61	6.244	0.000	0.60	4.817	0.000	0.60	6.878	0.000
21	–	–	–	0.61	5.359	0.000	0.63	6.459	0.000	0.61	6.167	0.000
22	–	–	–	0.62	8.31	0.000	0.63	9.318	0.000	0.59	3.908	0.001
23	–	–	–	0.59	5.115	0.000	0.62	5.958	0.000	0.60	5.812	0.000
24	–	–	–	0.63	4.662	0.000	0.61	9.138	0.000	0.59	4.133	0.000

^a^*N* = 24 seasons for each league position

^b^Test value = 0.5

**Table 4:** Spearman correlation between league table position and home advantage (HA)

Division	*N*	Correlation Coefficient	Sig. (2-tailed)
Premier League	480^a^	0.298	0.000
Championship	576 ^b^	0.228	0.000
League One	576 ^b^	0.195	0.000
League Two	576 ^b^	0.143	0.001

^a^20 teams × 24 seasons

^b^24 teams × 24 seasons

**Table 5:** Positional home advantage (HA) by team ability in the English Premier League (EPL) and English Football League (EFL)

Division	Ability	*N*	HA		One Sample T Test (Test Value = 0.5)		Test of Homogeneity of Variances		Robust Test of Equality of Means	
			Mean	Standard Deviation	t	Sig	Levene Statistic	Sig	Welch^e^	Sig
Premier League	High	120^a^	0.58	0.05	15.995	0.000	22.155	0.000	27.361	0.000
	Moderate	240^b^	0.61	0.08	22.212	0.000				
	Low	120^a^	0.64	0.10	15.594	0.000				
Championship	High	144^c^	0.57	0.05	16.244	0.000	18.547	0.000	19.900	0.000
	Moderate	288^d^	0.60	0.07	25.048	0.000				
	Low	144^c^	0.61	0.09	14.258	0.000				
League One	High	144 ^c^	0.57	0.06	14.672	0.000	8.201	0.000	15.393	0.000
	Moderate	288^d^	0.59	0.07	20.058	0.000				
	Low	144^c^	0.61	0.08	16.565	0.000				
League Two	High	144^c^	0.57	0.06	12.418	0.000	5.080	0.007	5.860	0.003
	Moderate	288^d^	0.58	0.08	18.271	0.000				
	Low	144^c^	0.60	0.09	13.095	0.000				

^a^5 teams per season × 24 seasons

^b^10 teams per season × 24 seasons

^c^6 teams per season × 24 seasons

^d^12 teams per season × 24 seasons

^e^Asymptotically F distributed

## Declarations

For this article no studies with human participants or animals were performed by any of the authors. All studies performed were in accordance with the ethical standards indicated in each case.
